# LMP1 Induces p53 Protein Expression via the H19/miR-675-5p Axis

**DOI:** 10.1128/spectrum.00006-22

**Published:** 2022-06-08

**Authors:** Jun Li, Yan Zhang, Lingling Sun, Song Liu, Menghe Zhao, Bing Luo

**Affiliations:** a Department of Pathogenic Biology, Qingdao University Medical College, Qingdao, China; b Department of Clinical Laboratory, Zibo Central Hospital, Zibo, China; c Pathology Department, The Affiliated Hospital of Qingdao University, Qingdao, China; d Municipal Centre of Disease Control and Prevention of Qingdao, Qingdao Institute of Prevention Medicine, Qingdao, Shandong Province, China; Oklahoma State University, College of Veterinary Medicine

**Keywords:** Epstein-Barr virus, latent membrane protein 1 (LMP1), p53, H19, miR-675-5p

## Abstract

Epstein-Barr virus (EBV), a ubiquitous oncogenic herpesvirus, infects more than 90% of the adult population worldwide. The long noncoding RNA H19 is downregulated in EBV-positive gastric cancer (EBVaGC) and nasopharyngeal cancer (NPC). In this study, we found that loss of H19 is caused by hypermethylation status of the H19 promoter in EBV-positive GC and NPC cell lines. Furthermore, latent membrane protein 1 (LMP1), encoded by EBV, induced H19 promoter hypermethylation and deregulated the expression of H19 by upregulating DNMT1 expression. Transwell assays showed that H19 promoted cell migration. Furthermore, H19 promoted cell proliferation and inhibited apoptosis in CCK-8 and flow cytometry assays, respectively. p53, a well-known tumor suppressor, was upregulated in EBVaGC and NPC cell lines. miR-675-5p derived from H19 inhibited p53 protein expression by targeting the 3′ untranslated region of the gene. Overall, we found that LMP1 induced p53 protein expression via the H19/miR-675-5p axis in EBVaGC and NPC. LMP1 induced H19 promoter hypermethylation, which repressed the expression of H19 and miR-675-5p and caused p53 protein overexpression in EBVaGC and NPC cells.

**IMPORTANCE** Epstein-Barr virus (EBV) is the first virus to be known to have direct association with human cancer and to be considered as an important DNA tumor virus. The EBV life cycle consists of both latent and lytic modes of infection in B lymphocytes and epithelial cells. The persistence of EBV genomes in malignant cells promoted cell growth. p53, acting as a critical gatekeeper tumor suppressor, is involved in multiple virus-mediated tumorigeneses. Overexpression of p53 inhibits the ability of BZLF1 (EBV-encoded immediate early gene) to disrupt viral latency. In our study, we found LMP1 induces H19 promoter hypermethylation, which represses the expression of H19 and miR-675-5p and results in p53 protein overexpression in EBVaGC and NPC cells. These observations suggest a new mechanism of aberrant expression of p53 by LMP1, which facilitates EBV latency.

## INTRODUCTION

Epstein-Barr virus (EBV), a ubiquitous gamma-herpes herpesvirus, infects more than 90% of the adult population worldwide (1). EBV was the first virus to be isolated from human tumor cells over 50 years ago. Recent studies have shown that EBV is closely associated with various human tumors including Burkitt’s lymphoma, nasopharyngeal carcinoma (NPC), Hodgkin’s lymphoma, Post-transplantation lymphoproliferative disorders (PTLDs), EBV-associated gastric cancer (EBVaGC), and others (2–4). The EBV life cycle consists of both latent and lytic modes of infection in B lymphocytes and epithelial cells. There are three known patterns of latent EBV infection that depend on the expression pattern of latent viral proteins (5, 6). EBV infection is a recognized epigenetic driver of tumorigenesis and may be involved in the extensive methylation induction of host gene promoter regions (7). Latent membrane protein 1 (LMP1), encoded by EBV, functions as a classic oncogene in rodent fibroblast transformation assays. It activates several downstream signaling pathways, such as the NF-κB, MAPK, and phosphatidylinositol 3-kinase/AKT pathways (8–11).

Long-chain noncoding RNAs (lncRNAs) are a type of noncoding RNA containing more than 200 nucleotides that regulate gene expression at the level of transcription, mRNA splicing, and translation (12). lncRNA H19, a 2.3-kb lncRNA, has recently been reported to regulate tumorigenesis. However, it is controversial as to whether H19 is a tumor suppressor or a tumor promoter (13). Genomic imprinting is a form of gene regulation in which genes are monoallelically expressed depending on the parental cell of origin. H19 and insulin-like growth factor 2 are imprinted in mammals. Loss of imprinting (LOI) may cause serious imprinting disorders and has been described in numerous cancers (14). H19 was reported to be a precursor of two distinct microRNAs (miRNAs), miR-675-5p and miR-675-3p. Furthermore, studies have shown that H19 and miR-675 are significantly upregulated in gastric cancer cells and tissues. This suggests that the H19/miR-675-5p axis plays an important role in tumorigenesis.

The p53 tumor suppressor is involved in virus-mediated tumorigenesis and diverse cellular stresses such as DNA damage and hypoxia (15–17). The relationship between EBV infection and p53 expression has been reported in various cancers including non-Hodgkin’s lymphoma of the head and neck, nasopharyngeal cancer, Burkitt’s lymphoma, and gastric carcinoma (18). Immunohistochemistry revealed that p53 accumulation is significantly correlated with LMP1 expression (19–21). Overexpression of p53 occurs at an early stage in the development of NPC and is associated with advanced stage disease and poor response to therapy (22–24). Furthermore, LMP1 disrupts p53-induced cell cycle arrest and apoptosis by modulating the K63-linked ubiquitination of p53 (22).

In the present study, we found that LMP1 induces H19 promoter hypermethylation by upregulating DNMT1 expression and deregulates the expression of H19. Furthermore, miR-675-5p derived from H19 inhibited p53 protein expression. Our study uncovers a new mechanism in which LMP1 induces p53 protein expression via the H19/miR-675-5p axis.

## RESULTS

### LMP1 downregulates H19 expression and induces the expression of p53 protein.

**FIG 1:**
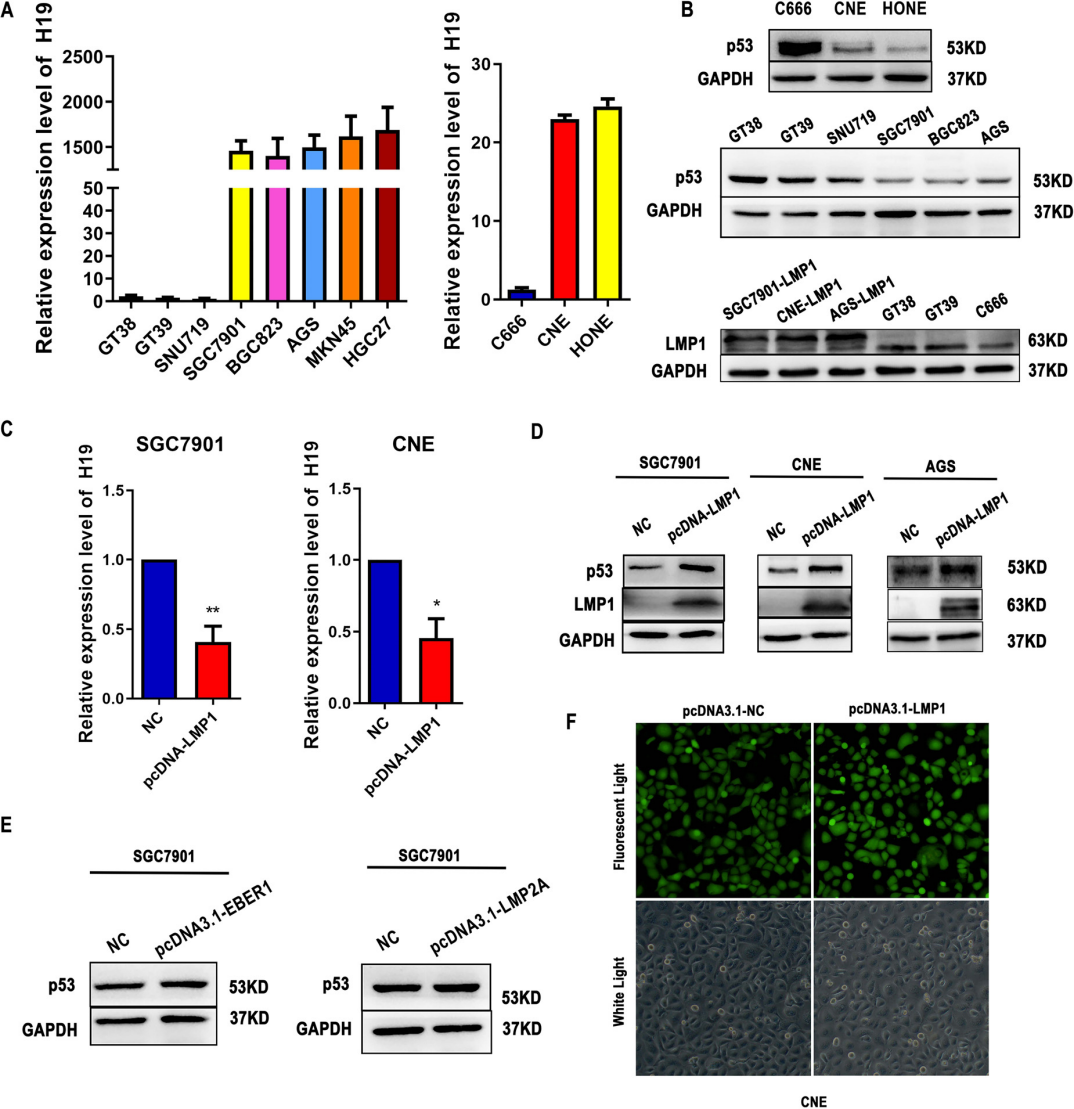
Expression of H19 and p53 in Epstein-Barr virus (EBV)-positive and EBV-negative tumor cell lines. (A) The expression of H19 mRNA in EBV-positive tumor cell lines (GT38, GT39, SNU719, and C666) and EBV-negative tumor cell lines (SGC7901, AGS, BGC823, MKN45, HGC27, CNE, and HONE) were measured by quantitative (q)RT-PCR. (B) Western blot analysis showing the protein levels of latent membrane protein 1 (LMP1) or p53 in EBV-positive tumor cell lines (GT38, GT39, SNU719, and C666) or EBV-negative tumor cell lines (SGC7901, AGS, BGC823, CNE, and HONE). (C) The expression of H19 mRNA in LMP1-transfected SGC7901 and CNE cells. (D) The expression of p53 protein in LMP1-transfected SGC7901 and CNE cells. (E) The expression of p53 protein in SGC7901 transfected with LMP2A and EBER1. (F) Fluorescence efficiency of CNE cells transfected with vector and LMP1 plasmids. NC, negative control cell. Original magnification × 400 for A to F. All experiments were performed with at least 3 replicates. *, *P < *0.05, **, *P < *0.01.

As shown in Fig. 1A and B, we compared the expression of H19 and p53 protein in EBV-positive and EBV-negative epithelial cancer cells. The results showed that H19 was highly expressed in EBV-negative compared with EBV-positive cancer cells. The p53 protein level was significantly higher in EBV-positive cancer cells. Furthermore, we detected the protein levels of LMP1 in EBV-positive cancer cells and transfected cells. To determine whether the EBV-encoded products can regulate the expression of H19 and p53 protein, EBV-negative cancer cells SGC7901, CNE or AGS were stably transfected with the LMP1, LMP2A, or EBV-encoded small RNA 1 (EBER1) gene. Following transfection, the level of H19 was significantly decreased in LMP1-transfected SGC7901 and CNE cells. Furthermore, the level of p53 protein was significantly upregulated in LMP1-transfected SGC7901, CNE and AGS cells (Fig. 1C and D). However, there was no difference in EBER1- and LMP2A-transfected SGC7901 cells (Fig. 1E). The stable transfected LMP1, LMP2A, and EBER1-SGC7901 cell lines were described previously (25). The EBV-negative NPC cell line CNE was stably transfected with LMP1 gene. Cells that successfully transfected express green fluorescent protein (Fig. 1F).

### Methylation of the H19 promoter in EBV-positive tumor cell lines.

**FIG 2:**
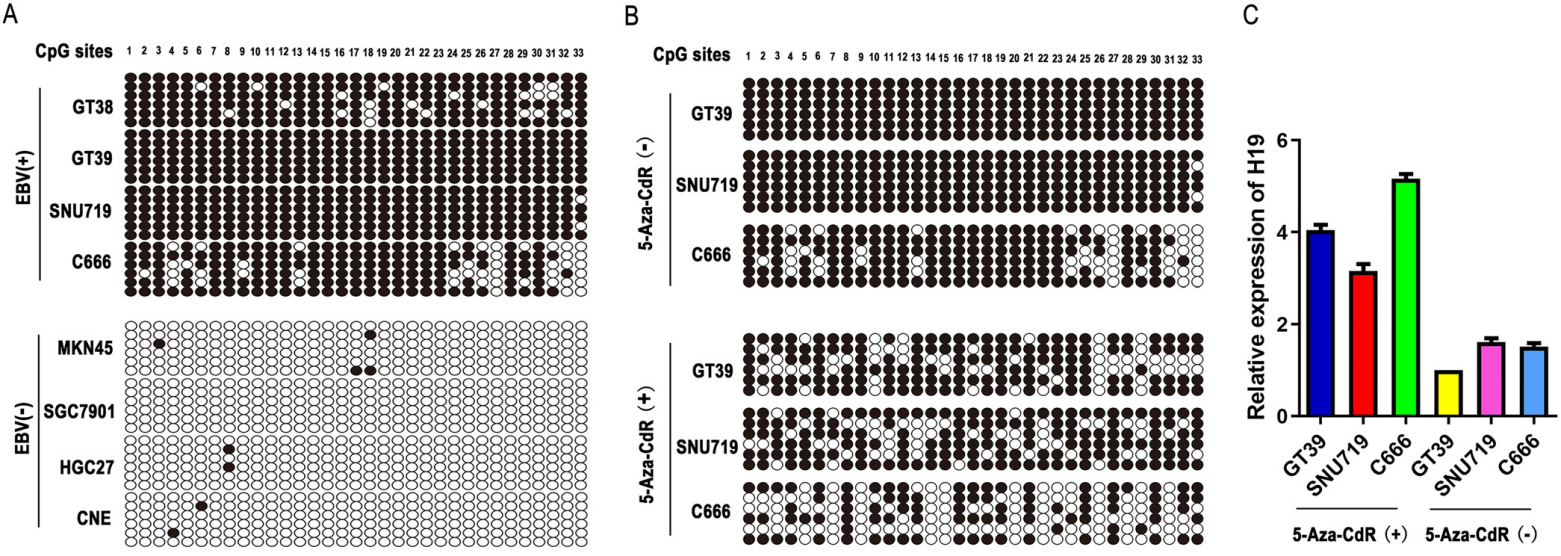
Methylation of the H19 promoter in EBV-positive and EBV-negative tumor cell lines. (A) The methylation status of CpG sites in H19 gene promoter in four EBV-positive and four EBV-negative tumor cell lines. Closed circles, methylated CG site; open circles, unmethylated CG site. (B) The methylation status of CpG sites in the H19 gene promoter in GT39, SNU719, and C666 cell lines after treatment with 5-Aza-CdR (15 μmol/L) for 3 days. (C) H19 mRNA expression in EBV-positive tumor cell lines (GT39, SNU719, and C666) were detected by quantitative (q)RT-PCR after treatment with 5-Aza-CdR (15 μmol/L) for 3 days. Expression levels were calculated by qRT-PCR. The GAPDH gene was used as internal reference and the 2^−△Ct^ method was used for relative quantification.

Bisulfite sequence PCR (BSP) was used to analyze the DNA methylation status of the H19 promoter region in GC and NPC cell lines. As shown in Fig. 2A, we observed a significantly higher methylation rate of the H19 promoter region in EBV-positive cancer cells compared with that in EBV-negative cancer cells. The methylation rates in the EBV-positive cancer cell lines GT38, GT39, SNU719, and C666, were 88.89%, 100%, 98.99%, and 75.76%, respectively, whereas in the EBV-negative cancer cell lines MKN-45, SGC7901, HGC27, and CNE, respectively, the methylation rates were 2%, 0, 1%, and 1%. Next, we determined the methylation status in EBV-positive cancer cell lines after treating with 5-Aza-CdR (a DNA methyltransferase inhibitor). BSP results showed that the methylation rates in the EBV-positive cancer cell lines, GT39, SNU719, and C666 were 71.21%, 74.24%, and 43.94%, respectively (Fig. 2B). The mRNA expression of H19 was higher in EBV-positive tumor cells treated with 5-Aza-CdR (15 μmol/L) for 3 days compared with EBV-positive tumor cells treated with DMSO.

### LMP1 induces DNMT1 upregulation and subsequent downexpression of H19 in EBVaGC and NPC cell lines.

**FIG 3:**
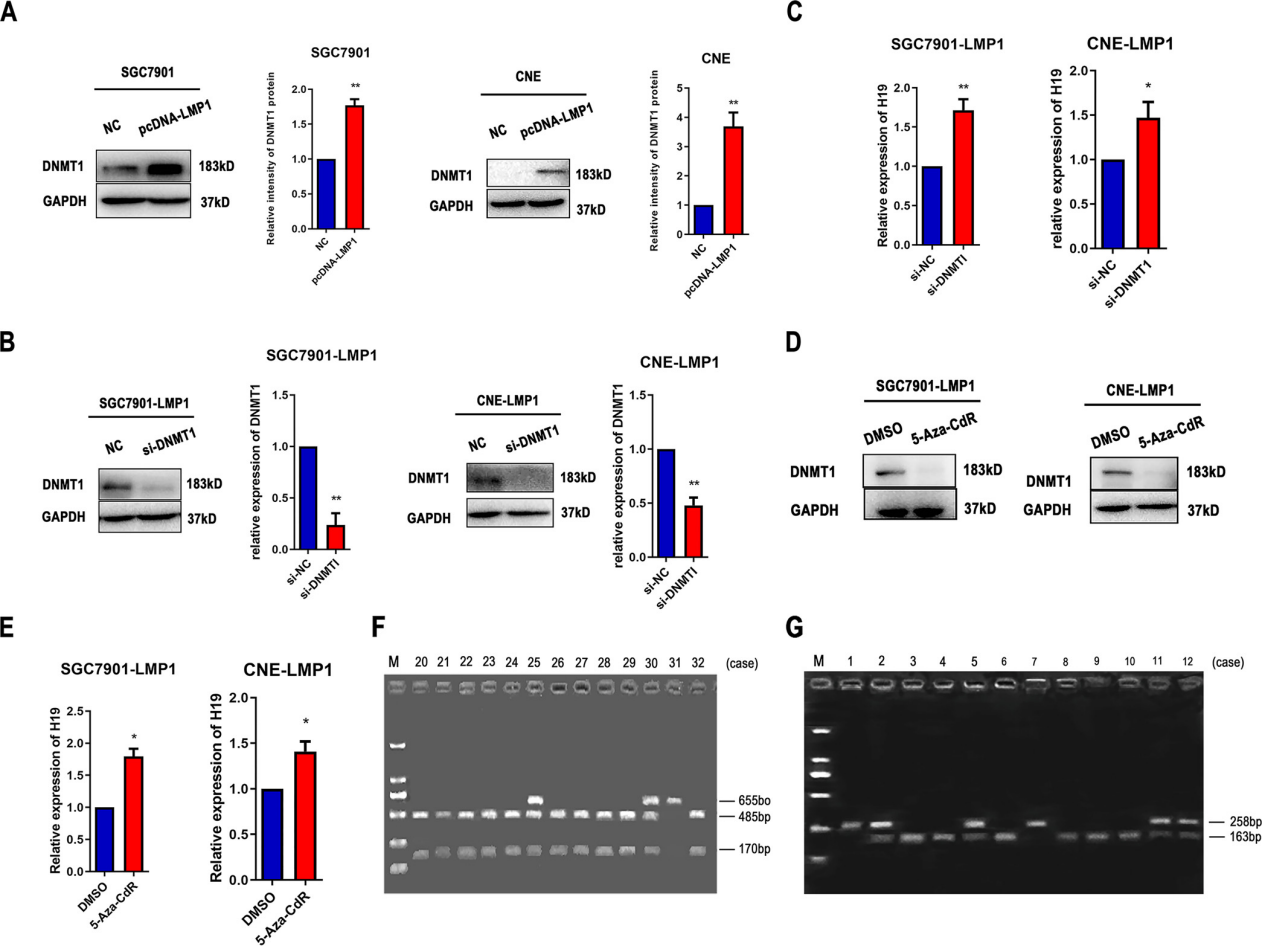
LMP1 reduces H19 mRNA expression by upregulating DNMT1 protein levels. (A) Protein levels of DNMT1 in LMP1-transfected SGC7901 and CNE cells. (B) Protein levels of DNMT1 in LMP1-transfected SGC7901 and CNE cells transfected with si-DNMT1 or si-NC. (C) H19 mRNA expression levels in LMP1-transfected SGC7901 and CNE cells transfected with si-DNMT1 or siNC. (D) Protein levels of DNMT1 in LMP1-transfected SGC7901 and CNE cells after treatment with 5-Aza-CdR (15 μmol/L) for 3 days. (E) H19 mRNA expression in LMP1-transfected SGC7901 and CNE cells after treatment with 5-Aza-CdR (15 μmol/L) for 3 days. (F) Analysis of H19 heterozygosity. RsaI digestion of a 655 bp PCR product yielded bands of 485 and 170 bp (cases 20–24, 26–29, and 32) or 655 bp (case 31) indicating homozygosity. Heterozygous specimens (cases 25 and 30) yielded all three bands. M, molecular weight marker. (G) Analysis of H19 imprinting status. RsaI-digested PCR product yielded bands of 258 bp (cases 1 and 7) or 163 bp (case 3, 4, 6, 8, 9, and 10) indicating maintenance of constitutional imprinting. LOI specimens (cases 2, 5, 11, 12) showed both bands. M, molecular weight marker. All experiments were performed with at least 3 replicates. *, *P < *0.05, **, *P < *0.01.

Western blot analysis revealed that DNMT1 was significantly upregulated in LMP1-transfected SGC7901 and CNE cells (*P < *0.01). To explore the effect of DNMT1 on H19, we inhibited the expression of DNMT1 protein using siRNA in LMP1-transfected SGC7901 and CNE cells (Fig. 3B). As shown Fig. 3C, downregulation of DNMT1 increased the expression of H19 as measured by quantitative (q)RT-PCR. The protein level of DNMT1 was reduced in LMP1-transfected cells treated with 5-Aza-CdR (15 μmol/L) for 3 days, whereas the expression of H19 was markedly upregulated (Fig. 3D and E).

### Allele-specific expression of H19 in EBV-positive GC, NPC, and lymphoma tissues.

The H19 gene is imprinted in mammals. LOI may lead to serious imprinting disorders and is associated with many cancers. In heterozygote samples, biallelic expression of H19 was suggested by LOI. To explore the imprinting status of H19, we detected allelic expression of H19 using agarose gel electrophoresis in EBVaGC, NPC, and lymphoma tissues (the representative bands are shown in Fig. 3F and G). Constitutive imprinting yielded either a single 258- or 163-bp bands. LOI resulted in 258- and 163-bp fragments. LOI of H19 was observed in lymphoma tissues (Table 1, *P < *0.05). However, it was not observed in EBV-positive GC and NPC tissues (Table 1, *P > *0.05).

**TABLE 1:** Allele-specific expression of H19 in EBV-positive GC, NPC, and lymphoma tissues^*a*^

Imprinted status	EBV (+)	EBV (−)	*P* value^*b*^
Imprinted status of H19 gene in EBVaGC and EBVnGC tissues			
	EBVaGC (*n* = 24)	EBVnGC (*n* = 28)	
LOI	6 (25.00%)	6 (21.43%)	*P* > 0.05
Normal imprinting	18 (75.00%)	22 (78.57%)	
Imprinted status of H19 gene in EBV-positive NPC and EBV-negative NPC tissues			
	EBV-positive NPC (*n* = 42)	EBV-negative NPC (*n* = 5)	
LOI	28 (66.67%)	2 (40.00%)	*P* > 0.05
Normal imprinting	14 (33.33%)	3 (60.00%)	
Imprinted status of H19 gene in EBV-positive lymphoma and EBV-negative lymphoma tissues			
	EBV-positive lymphoma (*n* = 36)	EBV-negative lymphoma (*n* = 30)	
LOI	22 (61.11%)	11 (36.67%)	*P* < 0.05
Normal imprinting	14 (38.89%)	19 (63.33%)	

a EBV, Epstein-Barr virus; EBVaGC, EBV-associated gastric carcinoma; EBVnGC, EBV-negative gastric; GC, gastric cancer; LOI, loss of imprinting; NPC, nasopharyngeal cancer.

b From chi-square or Fisher’s exact test.

### H19 promotes the proliferation of gastric cancer cells.

**FIG 4:**
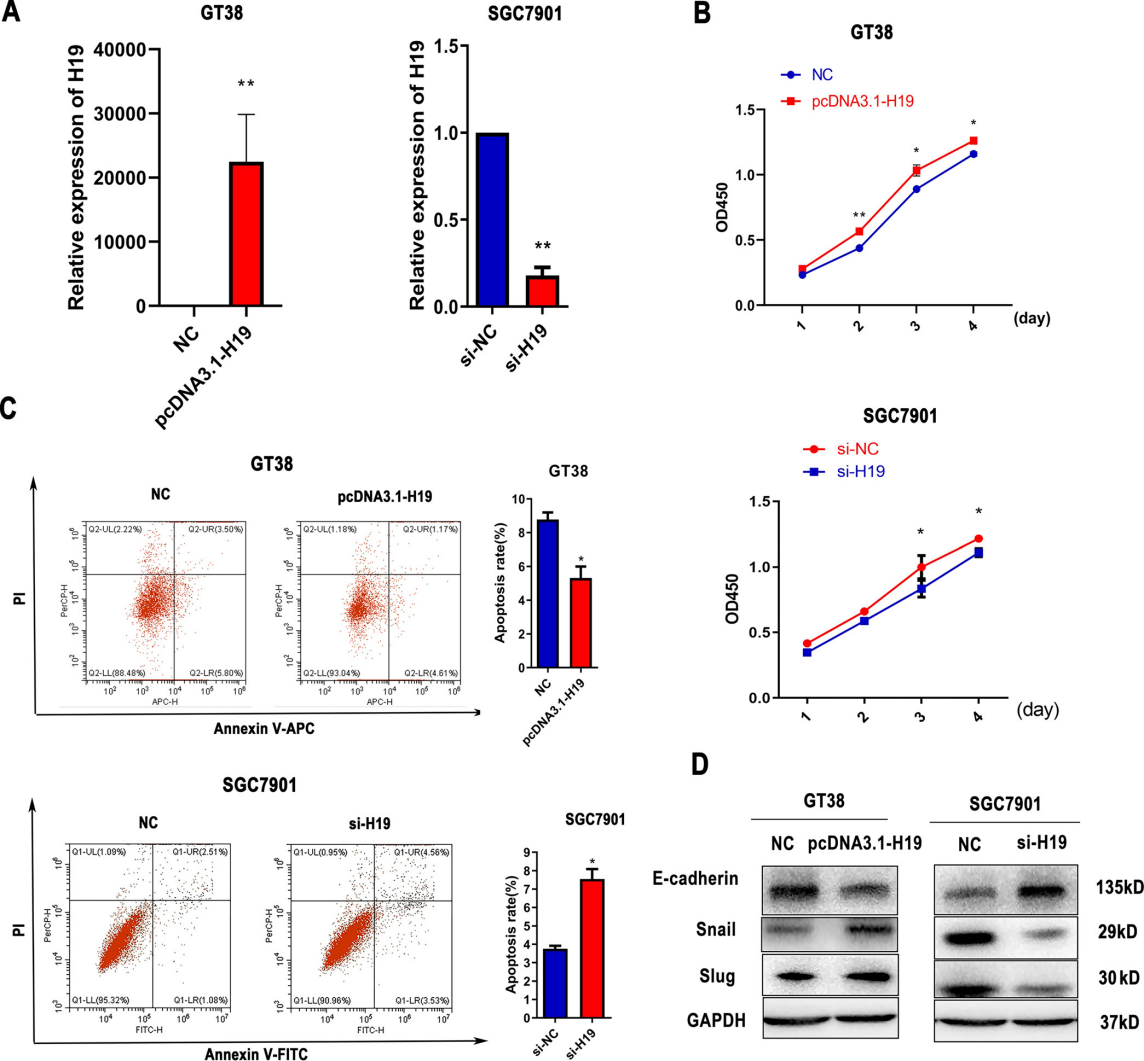
H19 promotes the proliferation of gastric cancer (GC) cells. (A) The transfection efficiency of si-H19 and H19 overexpressing plasmids in SGC7901 or GT38 cells. (B) Proliferation of GT38 and SGC7901 cells was detected by CCK-8 assay after transfection for 24, 48, 72, and 96 h. (C) The apoptosis rate was measured by flow cytometry in transfected SGC7901 and GT39 cells. (D) Expression of epithelial-mesenchymal transition (EMT)-related proteins in transfected GT38 cells and SGC7901 cells. All experiments were performed with at least three replicates. *, *P < *0.05, **, *P < *0.01.

To explore the function of H19 in GC cells, we suppressed H19 expression using siRNA and overexpressed H19 expression by transfecting a reporter plasmid (Fig. 4A). As shown in Fig. 4B, the proliferation of small interfering (si)-H19 cells was significantly lower compared with that of si-negative control (NC) cells at 72 and 96 h in SGC7901 cells, whereas overexpression of H19 yielded the opposite pattern in GT38 cells. Moreover, cell apoptosis notably decreased after overexpression of H19 in GT39 cells and increased after H19 suppression in SGC7901 cells (Fig. 4C). To determine whether H19 influences the epithelial-mesenchymal transition (EMT) process, we analyzed the expression of EMT-associated genes in transfected cells via Western bolt analysis. As shown in Fig. 4D, H19 reduced the expression of E-cadherin and increased the expression of Slug and Snail compared with the scrambled control.

### LMP1 induces the expression of p53 via the H19/miR-675-5p axis.

**FIG 5:**
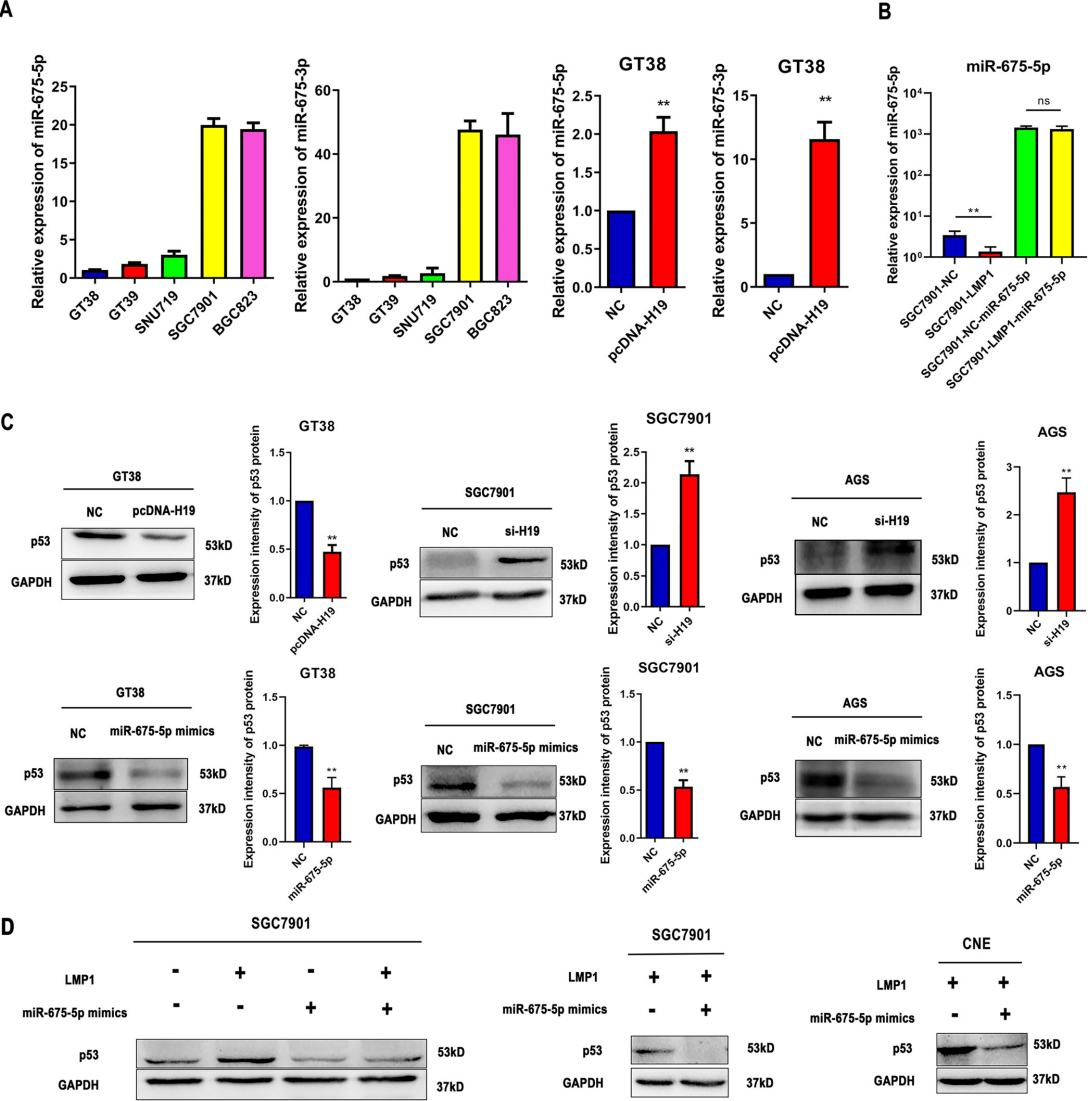
LMP1 induces the expression of p53 protein via the H19/miR-675-5p axis. (A) miR-675-5p and miR-675-3p expression levels in EBV-positive tumor cell lines (GT38, GT39, and SNU719), EBV-negative tumor cell lines (SGC7901, BGC823), and H19 overexpressed GT38 cells. (B) The expression levels of miR-675-5p in control transfected, LMP1 transfected and LMP1-miR-675-5p mimics transfected SGC7901 cells. (C) Protein levels of p53 in H19 overexpressing GT38 cells. The expression levels of p53 in AGS and SGC7901 cells transfected with si-H19 or miR-675-5p mimics. (D) Protein levels of p53 in SGC7901 cells transfected with miR-675-5p mimics or LMP1 plasmids. LMP1-transfected SGC7901 cells were transfected with miR-675-5p mimics or mimic NC for 48 h. The expression of p53 was detected by Western blot analysis. All experiments were performed with at least 3 replicates. **, *P < *0.01.

H19 was reported as a precursor for two distinct microRNAs (miRNAs), miR-675-5p and miR-675-3p. As shown in Fig. 5A, the expression of miR-675-5p and miR-675-3p was downregulated in EBVaGC cells. Furthermore, we detected miR-675-5p and miR-675-3p expression in H19-transfected GT38 cells indicating that they were upregulated in H19-transfected GT38 cells. Furthermore, the expression levels of mature miR-675-5p control-transfected, LMP1-transfected, and LMP1-miR-675-5p mimic-transfected SGC7901 cells were measured by qRT-PCR (Fig. 5B). As shown in Fig. 5C, Western blot analysis revealed decreased expression of H19 by siRNA and increased p53 protein levels in both SGC7901 and AGS cell lines. In contrast, overexpression of H19 decreased the expression of p53 protein in GT38 cell lines. Furthermore, overexpression of miR-675-5p resulted in a significant decrease in p53 protein levels 48 h posttransfection (*P < *0.01). LMP1 failed to induce p53 expression following transfection with miR-675-5p mimics. In addition, miR-675-5p significantly decrease p53 protein levels in LMP1-transfected SGC7901 and CNE cells (Fig. 5D).

### miR-675-5p promotes GC cell proliferation and migration by targeting p53.

**FIG 6:**
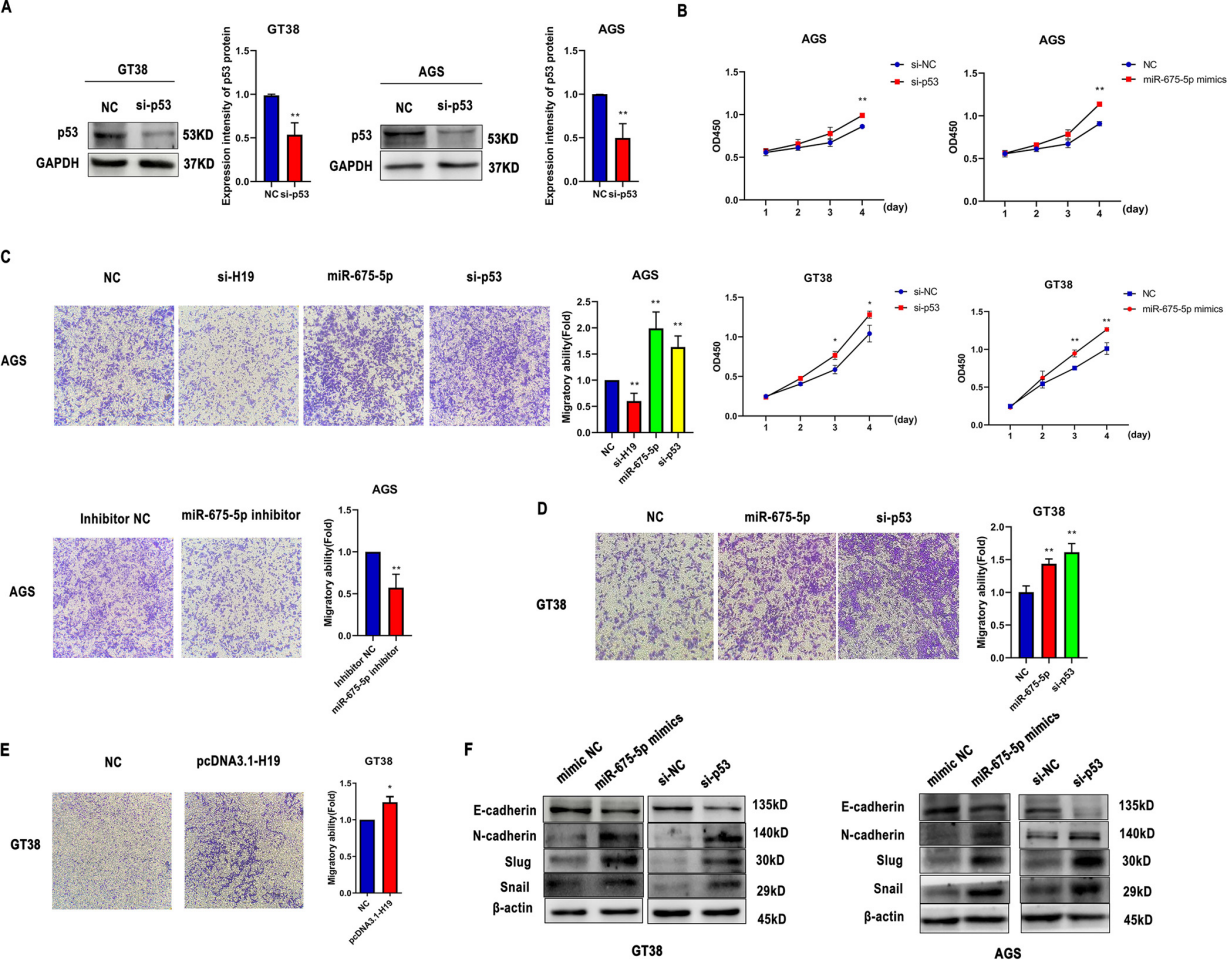
miR-675-5p promotes GC cell proliferation and migration by targeting p53. (A) The protein levels of p53 in GT38 and AGS cells transfected with si-p53 or si-NC. (B) Proliferation of GT38 and AGS cells transfected with si-p53 and miR-675-5p mimics was measured by CCK-8 assay for 24, 48, 72, and 96 h. (C-E) Effect of pcDNA3.1-H19, si-H19, miR-675-5p, si-p53, or miR-675-5p inhibitors on the migration ability of GT38 and AGS cells as detected by Transwell assay 48 h after transfection. (F) Expression of EMT-related proteins in GT38 and AGS cells transfected with miR-675-5p mimics and si-p53. All experiments were performed with at least 3 replicates. *, *P < *0.05, **, *P < *0.01.

To explore the function of p53 in GC cells, we suppressed p53 expression using siRNA (Fig. 6A). The proliferation of si-p53- and miR-675-5p-transfected AGS or GT38 cells was significantly higher compared with that of NC cells at 72 and 96 h (*P < *0.05) (Fig. 6B). As shown in Fig. 6C, D, and E, a Transwell assay was performed to evaluate the biological effects of H19, miR-675-5p, and si-p53 on cell migration. The results indicated that overexpression of H19 significantly promoted the migration ability of GT38 cells. Downregulation of H19 and miR-675-5p inhibited the migration of AGS cells. In contrast, si-p53 and miR-675-5p promoted cell migration. To determine whether miR-675-5p influences the EMT process, we analyzed the expression of EMT-associated genes in transfected cells via Western bolt analysis. In GT38 and AGS cells, miR-675-5p mimics and si-p53 reduced the expression of E-cadherin and increased the expression of N-cadherin, Slug, and Snail compared with the scrambled control (Fig. 6F).

## DISCUSSION

The latent membrane protein 1 (LMP1), a well-documented oncogene encoded by EBV, is translocated to the cell membrane and is expressed in most EBV-associated lymphoproliferative diseases and malignancies. The largest fraction of LMP1 is located in intracellular membranes where it is biologically active. Recent studies demonstrated that LMP1 induces DNA methylation by upregulating DNMT1 protein levels (26–28). In this study, we performed the first investigation of the methylation status of H19 in GC and NPC cell lines. The BSP results suggested that the methylation rates of the H19 promoter in EBV-positive cancer cells were significantly higher compared with that in EBV-negative cancer cells. Furthermore, the methylation rates of the H19 promoter in EBV-positive cancer cells were reduced following treatment with 5-Aza-CdR. As shown in Fig. 1A, the expression levels of H19 were lower in EBV-positive compared with EBV-negative cancer cells; however, the expression of H19 was significantly upregulated after treatment with 5-Aza-CdR in EBV-positive cancer cells (Fig. 2C). This suggests that EBV deregulates H19 expression by inducing H19 promoter methylation. We subsequently found that H19 expression was downregulated in LMP1-transfected SGC7901 and CNE cells compared with the negative control group. The expression of H19 was markedly upregulated in LMP1-transfected cells treated with 5-Aza-CdR or siDNMT1 (Fig. 3E). This indicates that LMP1 reduces H19 expression by upregulating DNMT1 protein levels.

Insulin-like growth factor 2 and H19 genes are imprinted in mammals, and H19 is transcribed from the maternal allele (29). Loss of H19 imprinting was associated with numerous disorders and may play an important role in the process that results in H19 overexpression. In several human cancers, both pediatric and adult, loss of imprinting (LOI) underlies gene expression. H19 lncRNA can be overexpressed via the LOI mechanism (30). In this study, we identified the H19 imprinting status in EBV-associated malignant tumor tissues. Our observations showed that LOI of H19 occurred in EBV-positive malignant lymphoma but was not observed in cancer of EBV-positive epithelial origin. It suggested that EBV induced LOI of H19 in malignant lymphoma. Although the precise mechanism of LOI of H19 on gene expression remains to be proven, it was confirmed that LOI of H19 is an organ-specific genetic change and that H19 overexpression may play an important role in the progression of esophageal and colorectal cancers (29). Therefore, EBV may promote the carcinogenic process of malignant lymphoma via regulating H19 expression and the exact mechanism remains to be revealed. Until now, there have been several controversial studies as to whether H19 is an oncogene or tumor suppressor gene (31). H19 overexpression has been reported in cancer cell lines and clinical specimens. Moreover, functional studies have verified the oncogenic effects of H19 (32). However, other studies have shown that H19 and miR-675 limited the growth of the placenta prior to birth and suppressed cell proliferation. Furthermore, H19/miR-675 repressed prostate cancer metastasis by targeting TGFBI (33, 34). In this study, we studied the biological function of H19 and miR-675-5p in GC cells and found that H19 and miR-675-5p promoted cell proliferation and migration. Zheng and colleagues (35) demonstrated that miR-675-5p inhibited p53 expression by targeting its 3′ untranslated region (UTR). Notably, p53 gene mutations mainly in exons 5 through 8 have been previously reported in various malignant carcinomas (36–38). Therefore, it is possible that miR-675-5p is also capable of suppressing mutant p53 expression assuming their 3′-UTR sequences are mutation free. In the present study, we found miR-675-5p also repressed the expression of p53 protein in both AGS (p53 wild-type) and SGC7901 (p53 mutant-type) cell lines. As known, one of the features of EBVaGC is the wild-type p53 (39). Then, we examined the biological function of p53 in AGS cells by suppressing the expression of p53 using siRNA. Consistent with the effect of miR-675-5p mimics, a reduction of p53 promoted cell proliferation and migration.

The EBV life cycle consists of both latent and lytic modes of infection in B lymphocytes and epithelial cells. The persistence of EBV genomes in malignant cells promoted cell growth. During latent infection, EBV genomes express EBV-encoded nuclear antigens (EBNA), latent membrane proteins (LMPs), EBV-encoded small RNAs (EBERs), and EBV-encoded BART microRNAs. Among these latent genes, LMP1 plays an important role in cell transformation and tumorigenesis (40). Furthermore, p53 has a pivotal part in maintaining EBV latency. Overexpression of p53 inhibits the ability of BZLF1 (EBV-encoded immediate early gene) to disrupt viral latency. During the EBV lytic cycle, transcription of BZLF1 is activated and p53 function is inhibited, which may ameliorate the inhibitory effect of p53 upon viral replication. Therefore, it is possible that LMP1 upregulates p53 to maintain EBV latent infection (22, 41). Li and colleagues (17) found that LMP1 promotes p53 accumulation by promoting K63-linked ubiquitination. In this study, we identified a new mechanism for LMP1 regulation of p53 in EBV-associated cancer cells. We identified that LMP1 upregulated p53 expression through the H19/miR-675-5p axis.

**FIG 7:**
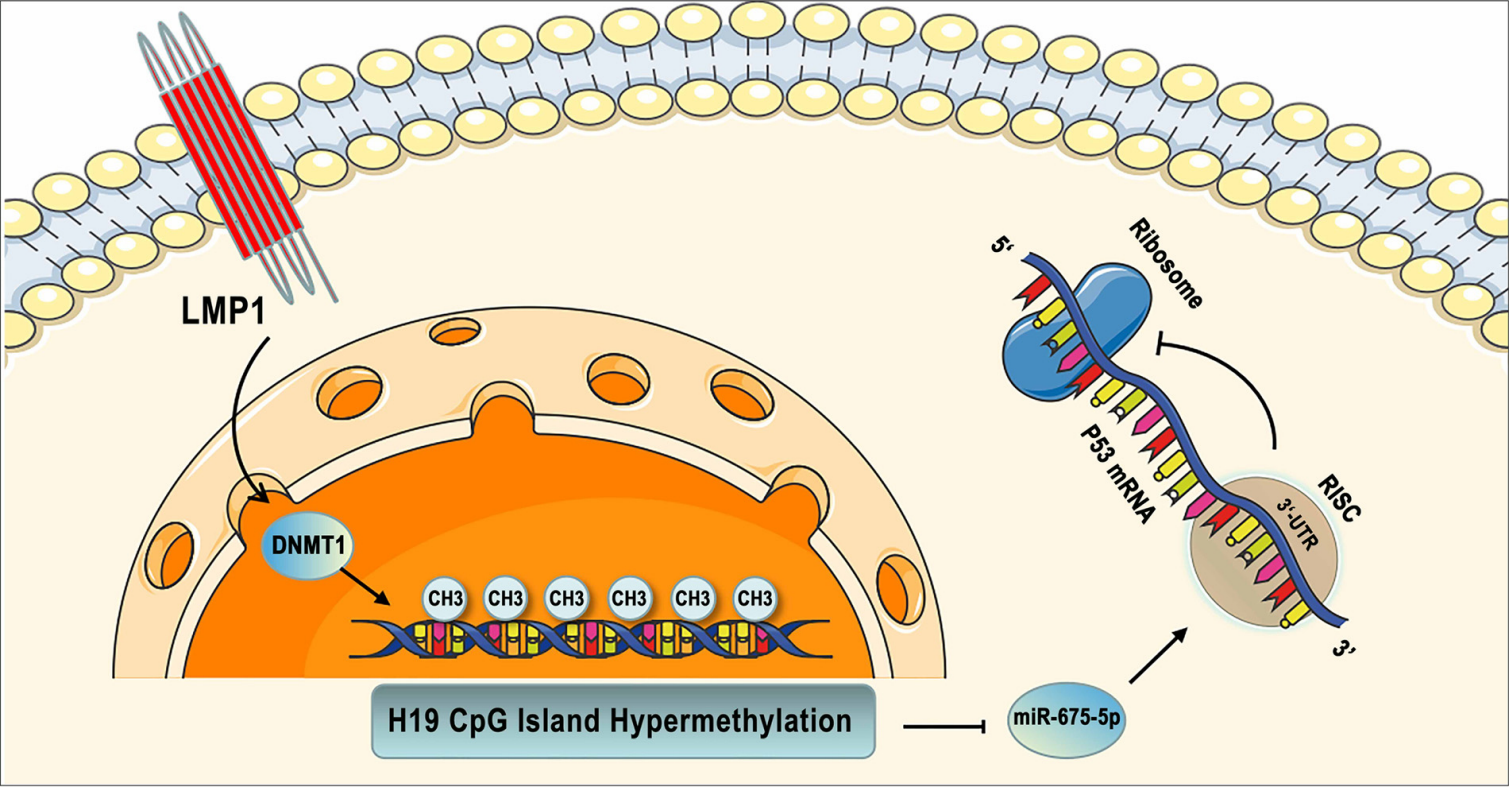
The model for LMP1-mediated p53 accumulation via the H19/miR-675-5p axis. LMP1 induced H19 CpG island hypermethylation via DNMT1. miR-675-5p was derived from H19 in gastric cancer and nasopharyngeal cancer cells. Decreased level of H19 was associated with low expression of miR-675-5p. miR-675-5p repressed expression of p53 protein by targeting its 3′ untranslated region. RISC, RNA-induced silence complex; UTR, untranslated region.

In summary, LMP1 induces H19 promoter hypermethylation, which represses the expression of H19 and miR-675-5p and results in p53 protein overexpression in EBVaGC and NPC cells (Fig. 7). These observations suggest a new mechanism of aberrant expression of p53 by LMP1 which facilitates EBV latency.

## MATERIALS AND METHODS

### Cell culture and reagents.

GT38, GT39, and SNU719 are EBVaGC cell lines. SGC7901, BGC823, AGS, and HGC27 are EBVnGC cell lines. C666 is a EBV-positive NPC cell line. HONE and CNE are EBV-negative NPC cell lines. The GT38 and GT39 cell lines were provided by T. Sairenji (Tottori University, Japan). The SNU719, CNE, and C666 cell lines were provided by Qian Tao (Chinese University of Hong Kong). The SGC7901, AGS, HGC27, BGC823, and HONE cell lines were procured from the Cell Culture Center (China).

All cell lines were maintained in DMEM supplemented with 10% fetal bovine serum in a humidified incubator containing 5% CO_2_ at 37°C. Paraffin-embedded and fresh tumor tissues were collected from the Affiliated Hospital of Qingdao University and Qingdao Municipal Hospital. This study received permission from the Medical Ethical Committee of Medical College of Qingdao University.

### Transfection with miR-675-5p inhibitor, mimics, and siRNA.

miR-675-5p inhibitor, mimics, negative control (NC), and siRNA were procured from Genepharma (China). As negative controls for the mimic and inhibitor, nonsense sequences were used. miR-675-5p mimics could mimic the role of miR-675-5p when used to transfect cells. Cells were seeded in 6-well plates at a density of 1 × 10^6^ per well and transfected with miR-675-5p inhibitor (100 nM), mimics (50 nM), NC (50 nM), or siRNA (50 nM) using Lipofectamine 2000 (Invitrogen, Carlsbad, CA, USA). The sequences were as follows: for mimics, 5′‐UGGUGCGGAGAGGGCCCACAGUG‐3′ and 5′‐CUGUGGGCCCUCUCCGCACCAUU‐3′; for miR-675-5p inhibitor, 5′‐CACUGUGGGCCCUCUCCGCACCA‐3′; for mimic NC, 5′‐UUCUCCGAACGUGUCACGUTT‐3′ and 5′‐ACGUGACACGUUCGGAGAATT‐3′; for inhibitor NC, 5′‐CAGUACUUUUGUGUAGUACAA‐3′; for sip53, 5′‐GAAUGAGGCCUUAGAGUUATT‐3′ and 5′‐UAACUCUAAGGCCUCAUUCTT‐3′; for si-H19, 5′‐AAGAAGCGGGUCUGUUUCUTT‐3′ and 5′‐AGAAACAGACCCGCUUCUUGC‐3′; and for si-NC, 5′‐UUCUCCGAACGUGUCACGUTT‐3′and 5′‐ACGUGACACGUUCGGAGAATT‐3′.

### Cell proliferation assay.

Cells were seeded at a density of 8 × 10^3^ per well in 96-well plates overnight. The cells were then transfected with miR-675-5p mimics, siRNA, or NC and incubated for 24, 48, 72, or 96 h. Cell viability was measured using Cell Counting Kit-8 (CCK-8, Solarbio) at the indicated times. Ten microliters of CCK-8 was added to each well, and after 1 h, the absorbance was measured at 450 nm using a microplate reader.

### Detection of apoptosis.

SGC7901 and GT38 cells were seeded in 6-well plates at a density of 1 × 10^6^ per well. SGC7901 cells were transfected with miR-675-5p mimics, siRNA, or NC for 48 h cells and then washed with cold PBS. Apoptosis was detected using an Annexin V-FITC Apoptosis Detection Kit (BD Biosciences, USA) according to the manufacturer’s instructions.

### Transwell cell migration assay.

Cell migration was measured using Transwell chambers with 8-μm pores (Corning, USA). Transwell assay was performed as described previously (42).The cells in five randomly selected fields were photographed under a microscope.

### RNA isolation and qRT-PCR.

Total RNA isolation and qRT-PCR were performed as described previously (43). The sequences of the specific forward and reverse primers were as follows: for H19, 5′-GCGGGTCTGTTTCTTTACTTC-3′ and 5′-TTTCATGTTGTGGGTTCTGG-3′; for GAPDH, 5′-CAAATTCCATGGCACCGTCA-3′ and 5′-ATCGCCCCACTTGATTTTGG-3′; for miR-675-5p, 5′‐ATTTGGTGCGGAGAGGGCCC‐3′; and for miR-675-3p, 5′‐CTGTATGCCCTCACCGCTCA‐3′

### Western blot analysis.

Western blot analysis was carried out as described previously (44). The following primary antibodies were used: anti-p53, anti-Snail, anti-β-actin, anti-GAPDH, anti-N-cadherin, and anti-Slug from Cell Signaling Technology (1:1,000); and anti-LMP1, anti-LMP2A, anti-EBNA1, anti-DNMT1, and anti-E-cadherin from Abcam (1:1,000).

### DNA extraction.

DNA extraction was carried out as described previously (25). The DNA from paraffin-embedded tumor tissues was extracted by the QIAamp DNA FFPE Tissue kit (Qiagen GmbH, Germany) according to the manufacturer’s instructions.

### DNA bisulfite treatment and methylation analysis.

Bisulfite modification of DNA was performed as described previously (25). The methylation status of the H19 CpG locus in GC and NPC cell lines was determined using BSP before and after the treatment with 5-Aza-CdR. The sequences of the BSP primers were as follows: 5′-AGATTTGAGGTGAATTTTAGGGATT-3′ and 5′-CCCTCAAAAAACACCATACCTACTA-3′.

### Genomic PCR amplification.

The sequences of the specific forward and reverse primers used to amplify DNA for the H19 gene were as follows: 5′-TACAACCACTGCACTACCTG-3′ and 5′-TGGAATGCTTGAAGGCTGCT-3′. The PCR system contained 2.5 μL of 10×Buffer, 1.5 mM MgCl_2_, 0.2 mM dNTPs, 0.3 μM upstream and downstream primer, 1.0 unit *Taq* DNA polymerase, and 0.5 μg DNA template or 3 μL of a 20-μL solution cDNA. Templates were subjected to PCR for 35 cycles. The cycling conditions for an initial denaturation at 94°C for 5 min, followed by denaturation at 94°C for 30 s, annealing at 72°C for 30 s, and extension at 72°C for 10 min.

### LOI detection.

For heterozygous samples, the corresponding RT-PCR products were restriction-digested with RsaI to assess the allelic expression of H19. The sequences of the specific forward and reverse primers were as follows: 5′-CCTCCACGACTCTGTTTCC-3′ and 5′-TGCTTGAAGGCTGCTCC-3′. Constitutive imprinting yielded either a single 258- or 163-bp band. LOI resulted in 258- and 163-bp fragments.

### Statistical analysis.

All data were analyzed using GraphPad Prism software version 5.0 (San Diego, CA, USA) and are expressed as the means ± SEM. Student's *t* test was used to analyze differences between groups. The correlation between the LOI or clinical characteristics and EBV expression was determined by Chi-square test. Difference with *P < *0.05 were considered statistically significant.

### Ethics approval and consent to participate.

All procedures performed in this study involving human participants were in accordance with the ethical standards of the Medical Ethics Committee at the Medical College of Qingdao University and with the Declaration of Helsinki 1964 and its later amendments or comparable ethical standards.
